# Plasma osteoprotegerin predicts adverse cardiovascular events in stable coronary artery disease: the PEACE trial

**DOI:** 10.3389/fcvm.2023.1178153

**Published:** 2023-06-14

**Authors:** Teng Ma, Jian Zhao, Yechao Yan, Junying Liu, Jie Zang, Yaqi Zhang, Kun Ruan, Hong Xu, Wan He

**Affiliations:** ^1^ Department of Cardiology, Shanghai Tenth People’s Hospital, Tongji University, Shanghai, China; ^2^ Emergency Department, Shanghai Tenth People's Hospital, Tongji University, Shanghai, China; ^3^ Department of Blood Transfusion, Shanghai Tenth People's Hospital, Tongji University, Shanghai, China

**Keywords:** coronary artery disease, biomarkers, osteoprotegerin (OPG), heart failure, prognostic model

## Abstract

**Background:**

Osteoprotegerin (OPG) is a secretory glycoprotein and participates in the progression of atherosclerotic lesions. We aim to explore the relationship between OPG and the prognosis of coronary artery disease (CAD).

**Methods:**

Plasma OPG concentrations were measured in 3,766 patients with stable CAD enrolled in the PEACE trial. The PEACE trial (NCT00000558) group followed up the patients and examined their future clinical outcomes.

**Results:**

In summary, 208 (5.5%) primary outcomes occurred, 295 patients (7.8%) died from all-cause death, 128 (3.4%) died from cardiovascular causes, and 94 (2.5%) experienced heart failure during a median follow-up of 1,892 days. In addition, we found that higher plasma levels of OPG were associated with a higher incidence of all-cause death, cardiovascular death, and heart failure, even after adjusting clinical cofounders.

**Conclusion:**

It was demonstrated that elevated plasma OPG levels were associated with an increased incidence of all-cause death, cardiovascular death, and heart failure in patients with stable CAD.

**Systematic Review Registration:**

https://clinicaltrials.gov/ct2/show/NCT00000558?term=NCT00000558&draw=2&rank=1, identifier: NCT00000558.

## 1. Introduction

Coronary artery disease (CAD) is a leading cause of morbidity and mortality around the world. Myocardial infarction (MI) or death is the first presentation in a significant number of patients with CAD. Despite improvements in technological and medical management of CAD treatment, the mortality rate is relatively high at present. Therefore, there is an urgent need to find a novel approach to identify a subset of patients at increased risk. A biomarker specific enough to allow for a non-invasive stratification of high-risk patients would be invaluable.

Osteoprotegerin (OPG), a cytokine belonging to the tumor necrosis factor (TNF) receptor family, has been identified as a resorption regulator (1). OPG is secreted by plenty of tissues, including the lung, kidneys, bone, immune tissues, cancer tissues, as well as cardiovascular system (heart and vascular tissues), as demonstrated by a series of studies (1–5). It is produced as a monomer (60 kD), a homodimer (120 kD), and also as a combination with its ligands—receptor activator of nuclear factor kappa B ligand (RANKL) and TNF-related apoptosis-inducing ligand (TRAIL) (6–8). Recent research has found that OPG may also play a role in the development of cardiovascular disease (CVD). Plasma OPG levels is significantly increased in both unstable angina (UA) and acute myocardial infarction (AMI) (9, 10). Moreover, high plasma concentrations of OPG are also associated with atherosclerotic lesions in the coronary arteries, which contributes to the prediction of cardiovascular mortality (11–15).

The PEACE trial enrolled patients with stable CAD and measured plasma OPG levels. In this study, we evaluated the prognostic potential of OPG for all-cause death and cardiovascular outcomes in a large cohort of patients with stable CAD.

## 2. Methods

### 2.1. PEACE study population

The PEACE study was a multicenter, randomized, placebo-controlled trial that enrolled 8,290 participants to assess the efficacy of angiotensin converting enzyme (ACE) inhibition in preventing cardiovascular events in patients with CAD (NCT number: NCT00000558). To be eligible, patients must additionally have serum creatinine levels ≤2.0 mg/dl, a left ventricular ejection fraction > 40%, and age ≥ 50 years. A comprehensive study description can be found elsewhere (16).

In this analysis, we used data from 3,766 PEACE participants who provided baseline blood samples with a measurement of OPG.

### 2.2. Clinical outcomes

The primary endpoint of our *post hoc* analysis was identified as a composite of cardiovascular death (CVD) or hospitalization for congestive heart failure (CHF). The secondary outcome included (1) all-cause death, (2) CVD, and (3) CHF.

### 2.3. Biomarker testing

Blood samples were collected in the clinical centers and routinely centrifuged at room temperature, then frozen within 90 min, and collected at −20°C. The samples were collected for 2 weeks (if clinics had a −20°C freezer) or 2 months (if clinics had a −70°C freezer) and shipped on dry ice to the PEACE central laboratory for −70°C storage for a long time.

Sandwich ELISA was used to measure plasma OPG, with commercially available antibodies (R&D Systems, Minneapolis, MN, USA) (17).

### 2.4. Statistical analysis

Baseline continuous variables were described using mean ± standard deviations (SD) or median (IQR). Comparisons were performed using the Mann–Whitney *U* test or one-way ANOVA test, respectively, between the three groups. The chi-squared test was used to assess the difference for categorical variables. The cumulative endpoint-free survival estimates were evaluated by the Kaplan–Meier method. We also evaluated the association between OPG and clinical outcomes in prognostic models, which includes the following variables: OPG, sex, estimated glomerular filtration rate (eGFR), total cholesterol, smoking, CCS functional classification, body mass index (BMI), and history of hypertension and diabetes. Schoenfeld's test was performed to confirm the proportional hazards assumption. All data were analyzed using IBM SPSS Statistics (version 25). Two-sided *P*-values < 0.05 were considered statistically significant.

## 3. Results

### 3.1. Characteristic at study entry

The baseline characteristics of patients with stable CAD are shown in Table 1. Among 3,766 patients with plasma OPG, the median OPG concentration was 2.2 ng/ml, 714 (19.0%) were female, and the average age was 64.1 years (mean ± 8.2). The median follow-up time was 1,892 days. Patients with higher OPG levels tended to be older and more likely to exhibit a higher systolic blood pressure (SBP), more severe angina (evaluated by CCS functional classification), and reduced renal function (assessed by eGFR). Meanwhile, the prevalence of hypertension, diabetes, and TIA increased in patients with stable CAD who had higher OPG levels, along with the proportion of patients who also used calcium channel blockers, diuretics, digitals, and anticoagulants. Patients with high OPG levels also had elevated levels of hs-TnT, C-reactive protein (CRP), NT-proBNP, and decreased eGFR. In contrast, higher plasma levels of OPG were negatively associated with the prevalence of MI and lipid-lowering therapy. OPG levels showed no correlation to BMI, LVEF, stroke, β blockers, and total cholesterol. As shown in Table 2 OPG has the strongest correlation with age (*r* = 0.426, *P* < 0.001), and also has a significant correlation with NT-proBNP (*r* = 0.237, *P* < 0.001) and TnT (*r* = 0.236, *P* < 0.001).

**Table 1:** Baseline characteristics for all patients and stratified according to the tertile of OPG.

OPG (ng/ml)					
Clinical characteristics	All (3,766)	OPG < 1.83 (1,248)	1.83 ≤ OPG < 2.60 (1,262)	OPG ≥ 2.60 (1,256)	*P*-value
OPG	2.2 (1.7–2.9)	1.5 (1.2–1.7)	2.2 (2.0–2.4)	3.3 (2.9–4.1)	–
Age, years	64.1 ± 8.2	60.0 ± 6.9	64.4 ± 7.6	68.1 ± 7.9	<0.001
Sex, female, *n* (%)	714 (19.0)	136 (10.9)	223 (17.7)	355 (28.3)	<0.001
BMI, kg/m^2^	28.5 ± 4.7	28.7 ± 4.5	28.4 ± 4.7	28.5 ± 5	0.417
SBP, mm/Hg	127 ± 17	124 ± 16	126 ± 17	130 ± 17	<0.001
DBP, mm/Hg	74 ± 10	75 ± 10	75 ± 10	74 ± 10	0.001
LVEF, %	59 ± 10	58 ± 10	59 ± 10	59 ± 10	0.089
**CCS functional classification**					<0.001
I	2,565 (68.1)	884 (70.8)	851 (67.4)	830 (66.1)	
II	768 (20.4)	230 (18.4)	263 (20.8)	275 (21.9)	
III	376 (10.0)	121 (9.7)	123 (9.7)	132 (10.5)	
IV	56 (1.5)	13 (1.0)	25 (2.0)	18 (1.4)	
**Medical history**					
Smoking, *n* (%)					0.001
Current	567 (15.1)	201 (16.1)	213 (16.9)	153 (12.2)	
Ever	2,295 (61.0)	759 (60.9)	777 (61.6)	759 (60.5)	
Never	901 (23.9)	287 (23.0)	272 (21.6)	342 (27.3)	
Hypertension, *n* (%)	1,685 (44.8)	496 (39.7)	555 (44.0)	634 (50.5)	<0.001
Diabetes mellitus, *n* (%)	616 (16.4)	119 (9.5)	203 (16.1)	294 (23.4)	<0.001
TIA, *n* (%)	124 (3.3)	23 (1.8)	50 (4.0)	51 (4.1)	0.002
Stroke, *n* (%)	161 (4.3)	44 (3.5)	55 (4.4)	62 (4.9)	0.212
Myocardial infarction, *n* (%)	2,114 (56.1)	763 (61.1)	726 (57.5)	625 (49.8)	<0.001
**Drug usage**					
β-blocker, *n* (%)	2,337 (62.1)	797 (63.9)	791 (62.7)	749 (59.7)	0.083
CCB, *n* (%)	1,257 (33.4)	372 (29.8)	417 (33.1)	468 (37.3)	<0.001
Potassium sparing diuretic, *n* (%)	124 (3.3)	28 (2.2)	40 (3.2)	56 (4.5)	0.008
Other diuretics, *n* (%)	346 (9.2)	65 (5.2)	110 (8.7)	171 (13.6)	<0.001
Digitalis, *n* (%)	123 (3.3)	19 (1.5)	42 (3.3)	62 (4.9)	<0.001
Lipid-lowering therapy, *n* (%)	2,707 (71.9)	954 (76.4)	911 (72.3)	842 (67.1)	<0.001
Antiplatelets, *n* (%)	3,432 (91.2)	1,161 (93.0)	1,158 (91.8)	1,113 (88.7)	<0.001
Anticoagulants, *n* (%)	178 (4.7)	42 (3.4)	50 (4.0)	86 (6.9)	<0.001
**Laboratory results**					
Total cholesterol, mg/dl	192.0 ± 39.0	191.6 ± 38.9	192.3 ± 38.2	192.1 ± 40.1	0.914
Potassium, mmol/L	4.3 ± 0.4	4.3 ± 0.4	4.3 ± 0.4	4.3 ± 0.4	0.033
eGFR, ml/min/1.73 m^2^	78 ± 19.4	81.4 ± 18.7	77.7 ± 18.8	74.8 ± 20.1	<0.001
TnT, ng/ml	0.006 (0.004–0.009)	0.005 (0.003–0.007)	0.006 (0.004–0.009)	0.007 (0.005–0.011)	<0.001
CRP, mg/L	1.7 (0.8–3.5)	1.4 (0.7–2.9)	1.7 (0.8–3.4)	2.1 (1.0–4.5)	<0.001
NT-proBNP, pg/ml	139 (71–272)	109 (58–208)	136 (69–264)	190 (91–365)	<0.001

BMI, body mass index; SBP, systolic blood pressure; DBP, diastolic blood pressure; LVEF, left ventricular ejection fraction; CCB, calcium channel blockers; TIA, transient cerebral ischemia; eGFR, estimate glomerular filtration rate; TnT, troponin T; CRP, C-reactive protein; NT-proBNP, N-terminal pro-B-type natriuretic peptide.

**Table 2:** Correlation analysis of OPG and clinical parameters.

	Age	BMI	SBP	DBP	eGFR	LVEF	Total cholesterol	Potassium	NT-proBNP	TnT	CRP
*r*	0.426	−0.028	0.164	−0.049	−0.177	0.027	0.01	0.056	0.237	0.236	0.173
*P* value	<0.001	0.092	<0.001	0.003	<0.001	0.101	0.528	0.001	<0.001	<0.001	<0.001
*N*	3,766	3,747	3,762	3,762	3,759	3,662	3,615	3,757	3,758	3,675	3,755

### 3.2. Baseline OPG levels and clinical events

**Figure 1:**
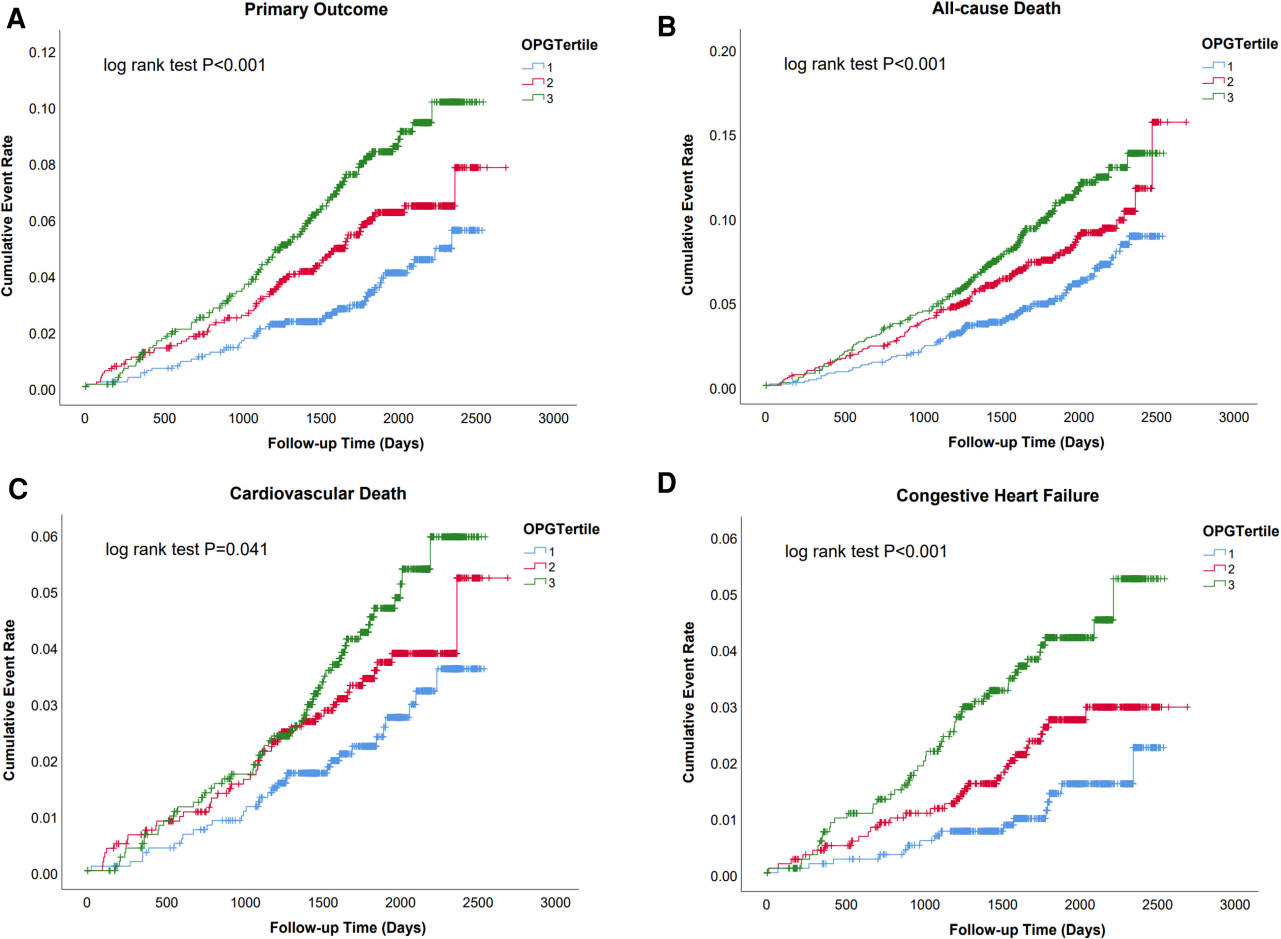
Survival regarding the primary endpoint (**A**. Cardiovascular death and congestive heart failure) and the secondary endpoint (**B**. All cause death; **C**. Cardiovascular death; **D**. Congestive heart failure) stratified according to the tertile distribution of OPG.

Over a median follow-up of 1,892 days (25th–75th percentiles 1,569–2,182), 208 (5.5%) cases of primary outcomes, 295 (7.8%) all-cause deaths, 128 (3.4%) cardiovascular deaths, and 94 (2.5%) heart failure events occurred. Kaplan–Meier analysis revealed a significantly lower possibility of developing clinical events in patients within the lowest tertiles of OPG levels (OPG < 1.83 ng/ml) (as shown in Figure 1 and Supplementary Figure 1).

**Figure 2:**
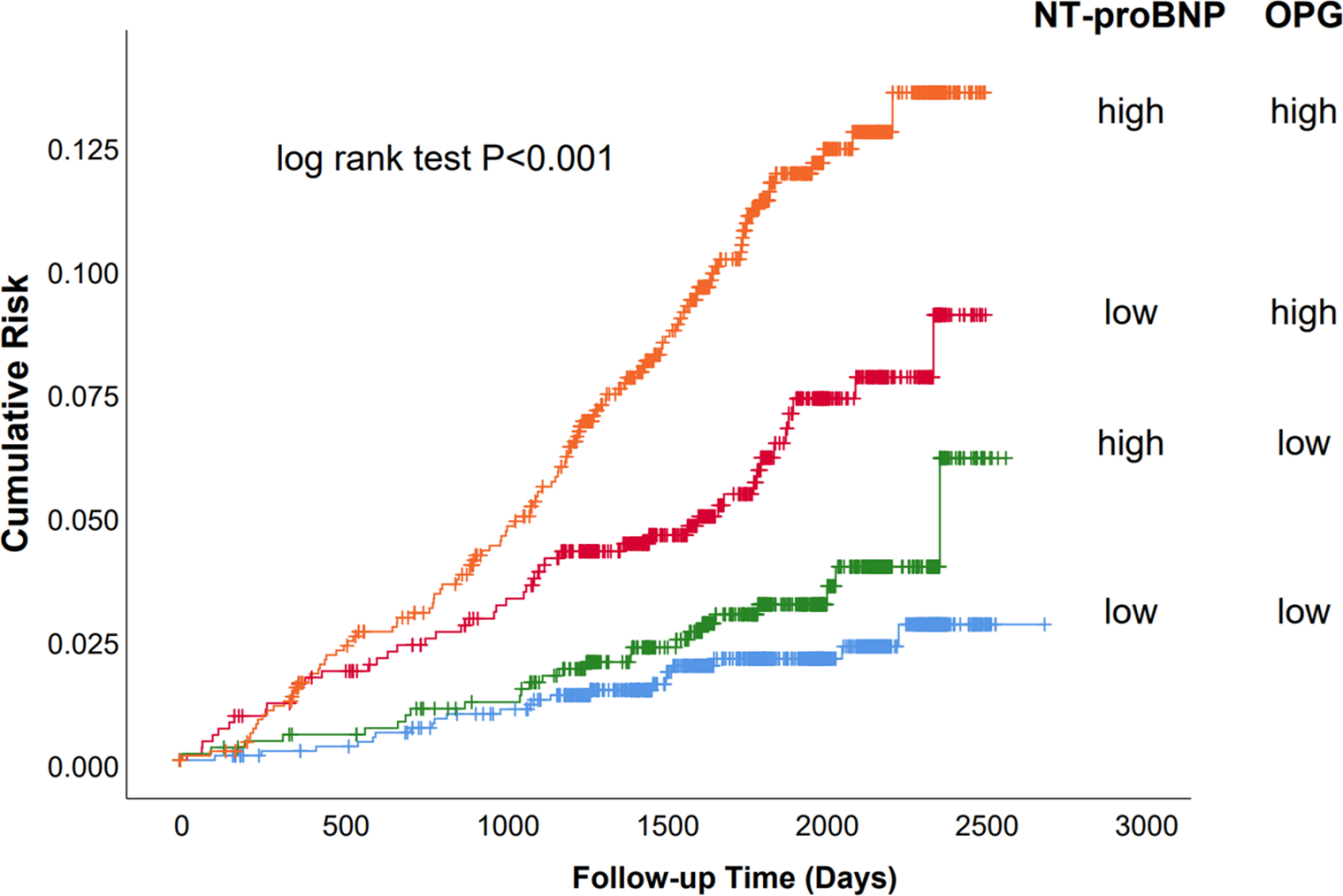
Survival of the primary endpoint (cardiovascular death and congestive heart failure) stratified by median baseline OPG and NT-proBNP levels. Kaplan–Meier analysis stratified by median baseline OPG and NT-proBNP levels. Baseline median OPG was 2.18 ng/ml; the median level for NT-proBNP was 139 pg/ml. “High” indicates values above the median and “low” values below the median.

The primary outcome was significantly different between patients with high (>2.18 ng/ml) and low (<2.18 ng/ml) OPG levels (Figure 2; log-rank test *P* < 0.001) in those with baseline NT-proBNP levels below the cohort median (<139 pg/ml). In this subset, those with high OPG values had a 6.0% incidence of the primary outcome, whereas those with low OPG values had a 2.0% incidence of adverse events. Similarly, in patients with high NT-proBNP levels, the rates of primary outcome were10.2% and 3.2% for OPG levels higher and lower than the median, respectively. Therefore, plasma OPG levels may further discriminate the risk of adverse outcomes on the basis of NT-proBNP measurement. cTnT is another significant biomarker of cardiac damage that is commonly used in clinical practice. We also found that the combination of OPG and cTnT could further differentiate patients at risk (Supplementary Figure S2).

A significant relationship between OPG and clinical endpoints was found using univariate and multivariable analysis with continuous and categorical OPG levels, even after adjustment in clinical models (as shown in Table 3 and Supplementary Table 1). The unadjusted model indicated that higher OPG levels were associated with a higher incidence of the primary outcome [HR: 2.20; 95% CI: (1.54,3.14); *P* < 0.001], all-cause death [HR, 1.81; 95% CI, (1.35,2.43); *P* < 0.001], cardiovascular death [HR, 1.75; 95% CI, (1.13,2.73); *P* = 0.01], and HF hospitalization [HR: 3.05; 95% CI: (1.73,5.36)]. After adjusting for clinical covariates, OPG levels were still related to primary outcome [HR: 1.75; 95% CI: (1.19,2.56); *P* < 0.001], all-cause death [HR: 1.69; 95% CI: (1.23,2.31); *P* < 0.001], cardiovascular death [HR: 1.62; 95% CI: (1.02,2.59); *P* = 0.04], and HF hospitalization [HR: 1.95; 95% CI: (1.06,3.56); *P* = 0.03]. We have explored the interactive effect of OPG and different medical history, including myocardial infarction, diabetes, hypertension, stroke, ACEI treatment (as shown in Supplementary Table 2 and Supplementary Table 3). And we found no difference between different groups.

**Table 3:** Associations between OPG and clinical outcomes, with adjustment for clinical characteristics.

	OPG < 1.83	1.83 ≤ OPG < 2.60	OPG ≥ 2.60
**Primary outcome**			
Number of events	44	69	95
Event rate (%)	3.5	5.5	7.6
Unadjusted HR (95% CI) (overall *P* < 0.001)	Ref	1.54 (1.06,2.25)	2.20 (1.54,3.14)
Adjusted HR (95% CI) (overall *P* < 0.001)	Ref	1.28 (0.87,1.90)	1.75 (1.19,2.56)
**All-cause death**			
Number of events	70	100	125
Event rate (%)	5.6	7.9	10.0
Unadjusted HR (95% CI) (overall *P* < 0.001)	Ref	1.40 (1.03,1.89)	1.81 (1.35,2.43)
Adjusted HR (95% CI) (overall *P* < 0.001)	Ref	1.28 (0.93,1.76)	1.69 (1.23,2.31)
**Cardiovascular death**			
Number of events	31	43	54
Event rate (%)	2.5	3.4	4.3
Unadjusted HR (95% CI) (overall *P* = 0.01)	Ref	1.35 (0.85,2.15)	1.75 (1.13,2.73)
Adjusted HR (95% CI) (overall *P* = 0.04)	Ref	1.20 (0.74,1.94)	1.62 (1.02,2.59)
**HF hospitalization**			
Number of events	16	30	48
Event rate (%)	1.3	2.4	3.8
Unadjusted HR (95% CI) (overall *P* < 0.001)	Ref	1.84 (1.01,3.38)	3.05 (1.73,5.36)
Adjusted HR (95% CI) (overall *P* = 0.03)	Ref	1.39 (0.74,2.58)	1.95 (1.06,3.56)

## 4. Discussion

The PEACE trial was the largest ever study evaluating the prognostic effect of OPG, and the main results of the present study show that elevated plasma OPG levels hold a long-term independent association with a composite outcome and all-cause death in patients with stable CAD, even after adjusting for the standard predictors. When attempting to interpret these findings, it is essential to note that an increase in plasma OPG might be a response to vascular calcification or atherosclerosis rather than a cause, perhaps representing an attempt to regulate the processes.

OPG is produced in a variety of organs, such as the intestines, kidneys, stomach, and bones, and is also secreted by plenty of cell types originating from the extracellular matrix, the immune system, and vessel wall, such as vascular smooth muscle cells (VSMCs) and vascular endothelial cells (ECs) (8, 18). It has been demonstrated that OPG participates in the metabolism and functions of ECs, and OPG also enhances the proangiogenic effect of VEGF and protects ECs from apoptosis (7, 19). In the ApoE−/− mice model, OPG knock-out was associated with an increased development of atherosclerosis (20). Inflammation and endothelial dysfunction play a central role in vascular disease and heart failure. Therefore, this suggests that OPG may play a pivotal role in the development of CAD and may even serve as a diagnostic or prognostic marker of CAD.

OPG was increased in various cardiovascular diseases such as peripheral artery disease (PAD), acute coronary syndrome (ACS), stable CAD, chronic heart failure, and so on (14). In a cohort of patients with CAD, the high plasma concentrations of OPG are connected with a wider variety of atherosclerotic lesions in the coronary arteries and an increased risk of death (6, 8, 15, 21). In a recent study, the investigators found that OPG holds long-term predictive power for all-cause mortality and cardiovascular events in patients with CAD. In addition, they found that OPG levels correlated positively with age, female sex, CRP, and diabetes status, but negatively with statin use (12). This indicates that OPG may be closely linked to metabolic status. It has been demonstrated that high circulating concentrations of OPG play a role in predicting the increased frequency of HF hospitalizations with a reduced ejection fraction (8). Previous research showed that the serum OPG level is a predictor of mortality and heart failure development in patients with AMI (22–24). Patients with stable coronary artery disease usually have impaired endothelial function. We found that a higher plasma OPG level was associated with adverse clinical events. In addition, different from NT-proBNP and cTnT, which are associated with cardiac pressure and/or volume load and myocardial injury, respectively, OPG is closely associated with endothelial function, and impairment of the endothelium triggers further cardiovascular events underpinning coronary artery disease. Interestingly, we found that OPG, in combination with either NT-proBNP or cTnT, significantly improved the predictive efficacy of the respective groups, allowing for further classification of the population.

In another study, baseline OPG and the frequency of low ejection fraction value were observed to be significantly correlated. At the same time, OPG predicted mortality but not the development of heart failure (25). The lack of statistical power in this analysis may be caused by the limited number of patients with HF. Omland et al. discovered that high levels of plasma OPG were correlated with elevated LV end-systolic volume and reduced LV ejection fraction, even after adjusting for clinical cofounders (26). However, we found there was no significant correlation between OPG and LVEF. Ejection fraction is not a good indicator of cardiac function and its limitations have become increasingly evident, especially among patients with HFpEF, the incidence of which has been increasing annually in recent years. Furthermore, OPG is closely related to endothelial function, and endothelial dysfunction is one of the pathological mechanisms of HFpEF. On the other hand, the inclusion criteria for the PEACE study were patients with an ejection fraction of 45% or more, and therefore, a population selection bias may have occurred. At the same time, in accordance with previous studies, a significant association was found between higher OPG levels and the incidence of heart failure hospitalization in the PEACE trial. All in all, OPG is a promising marker for predicting the prognosis of cardiovascular diseases.

## 5. Limitations

There are also some limitations in this study. First, the absence of serial measurements of OPG makes it impossible to evaluate the association between disease progression and levels of OPG. In addition, the concentration of OPG may be mediated by many factors, such as statin usage, which was ignored in the analysis. Then, the imbalance in sex proportions among the patients in the study may influence the levels of OPG.

## 6. Conclusion

The present study shows that OPG holds a long-term independent association with all-cause death, cardiovascular death, and heart failure hospitalization in patients with stable CAD. A combination of OPG and other clinical biomarkers could further pave the way for the formulation of precision interventions that target the different pathways involved. The accuracy and efficiency of OPG for diagnosis and prognosis, especially in patients with other cardiovascular diseases, may be worthy of investigation in the future.

## Data Availability

The original contributions presented in the study are included in the article/**Supplementary Materials**, and further inquiries can be directed to the corresponding authors.

## Supplementary material

Supplementary Figure 1 Survival regarding the myocardial infarction (A) and stroke (B) stratified according to the tertile distribution of OPG.

Supplementary Figure 2 Survival to the primary endpoint (cardiovascular death and congestive heart failure) stratified by median baseline levels of OPG and TnT. Kaplan-Meier analysis stratified by median baseline OPG and TnT levels. Baseline median OPG was 2.18 ng/mL; the median level for TnT was 5.97 ng/mL. “High” indicates values above the median and “low” values below the median.

## References

[1] Simonet WS Lacey DL Dunstan CR Kelley M Chang MS Lüthy R Osteoprotegerin: a novel secreted protein involved in the regulation of bone density. Cell. (1997) 89:309–19. 10.1016/s0092-8674(00)80209-3 9108485

[2] Yun TJ Chaudhary PM Shu GL Frazer JK Ewings MK Schwartz SM OPG/FDCR-1, a TNF receptor family member, is expressed in lymphoid cells and is up-regulated by ligating CD40. J Immunol. (1998) 161:6113–21. 10.4049/jimmunol.161.11.6113 9834095

[3] Hofbauer LC Shui C Riggs BL Dunstan CR Spelsberg TC O'Brien T Effects of immunosuppressants on receptor activator of NF-kappaB ligand and osteoprotegerin production by human osteoblastic and coronary artery smooth muscle cells. Biochem Biophys Res Commun. (2001) 280:334–9. 10.1006/bbrc.2000.4130 11162519

[4] Reid P Holen I. Pathophysiological roles of osteoprotegerin (OPG). Eur J Cell Biol. (2009) 88:1–17. 10.1016/j.ejcb.2008.06.004 18707795

[5] Wang Y Liu Y Huang Z Chen X Zhang B. The roles of osteoprotegerin in cancer, far beyond a bone player. Cell Death Discov. (2022) 8:252. 10.1038/s41420-022-01042-0 35523775 PMC9076607

[6] Bernardi S Bossi F Toffoli B Fabris B. Roles and clinical applications of OPG and TRAIL as biomarkers in cardiovascular disease. Biomed Res Int. (2016) 2016:1752854. 10.1155/2016/1752854 27200369 PMC4856888

[7] Rochette L Meloux A Rigal E Zeller M Cottin Y Vergely C. The role of osteoprotegerin and its ligands in vascular function. Int J Mol Sci. (2019) 20(3):705. 10.3390/ijms20030705 PMC6387017 30736365

[8] Montagnana M Lippi G Danese E Guidi GC. The role of osteoprotegerin in cardiovascular disease. Ann Med. (2013) 45:254–64. 10.3109/07853890.2012.727019 23110639

[9] Secchiero P Corallini F Beltrami AP Ceconi C Bonasia V Di Chiara A An imbalanced OPG/TRAIL ratio is associated to severe acute myocardial infarction. Atherosclerosis. (2010) 210:274–7. 10.1016/j.atherosclerosis.2009.11.005 20015493

[10] Erkol A Oduncu V Pala S Kızılırmak F Kılıcgedik A Yılmaz F Plasma osteoprotegerin level on admission is associated with no-reflow phenomenon after primary angioplasty and subsequent left ventricular remodeling in patients with acute ST-segment elevation myocardial infarction. Atherosclerosis. (2012) 221:254–9. 10.1016/j.atherosclerosis.2011.12.031 22265272

[11] Pedersen ER Ueland T Seifert R Aukrust P Schartum-Hansen H Ebbing M Serum osteoprotegerin levels and long-term prognosis in patients with stable angina pectoris. Atherosclerosis. (2010) 212:644–9. 10.1016/j.atherosclerosis.2010.06.027 20621297

[12] Bjerre M Hilden J Winkel P Jensen GB Kjøller E Sajadieh A Serum osteoprotegerin as a long-term predictor for patients with stable coronary artery disease and its association with diabetes and statin treatment: a CLARICOR trial 10-year follow-up substudy. Atherosclerosis. (2020) 301:8–14. 10.1016/j.atherosclerosis.2020.03.030 32289619

[13] Bjerre M. Osteoprotegerin (OPG) as a biomarker for diabetic cardiovascular complications. Springerplus. (2013) 2:658. 10.1186/2193-1801-2-658 24349960 PMC3863400

[14] Özkalaycı F Gülmez Ö Uğur-Altun B Pandi-Perumal SR Altun A. The role of osteoprotegerin as a cardioprotective versus reactive inflammatory marker: the chicken or the egg paradox. Balkan Med J. (2018) 35:225–32. 10.4274/balkanmedj.2018.0579 29687784 PMC5981118

[15] Røysland R Bonaca MP Omland T Sabatine M Murphy SA Scirica BM Osteoprotegerin and cardiovascular mortality in patients with non-ST elevation acute coronary syndromes. Heart. (2012) 98:786–91. 10.1136/heartjnl-2011-301260 22373720 PMC3341671

[16] Braunwald E Domanski MJ Fowler SE Geller NL Gersh BJ Hsia J Angiotensin-converting-enzyme inhibition in stable coronary artery disease. N Engl J Med. (2004) 351:2058–68. 10.1056/NEJMoa042739 15531767 PMC2556374

[17] Knudsen ST Foss CH Poulsen PL Andersen NH Mogensen CE Rasmussen LM. Increased plasma concentrations of osteoprotegerin in type 2 diabetic patients with microvascular complications. Eur J Endocrinol. (2003) 149:39–42. 10.1530/eje.0.1490039 12824864

[18] Corallini F Rimondi E Secchiero P. TRAIL And osteoprotegerin: a role in endothelial physiopathology? Front Biosci. (2008) 13:135–47. 10.2741/2665 17981533

[19] Malyankar UM Scatena M Suchland KL Yun TJ Clark EA Giachelli CM. Osteoprotegerin is an alpha vbeta 3-induced, NF-kappa B-dependent survival factor for endothelial cells. J Biol Chem. (2000) 275:20959–62. 10.1074/jbc.C000290200 10811631

[20] Bennett BJ Scatena M Kirk EA Rattazzi M Varon RM Averill M Osteoprotegerin inactivation accelerates advanced atherosclerotic lesion progression and calcification in older ApoE-/- mice. Arterioscler Thromb Vasc Biol. (2006) 26:2117–24. 10.1161/01.ATV.0000236428.91125.e6 16840715

[21] Shin JY Shin YG Chung CH. Elevated serum osteoprotegerin levels are associated with vascular endothelial dysfunction in type 2 diabetes. Diabetes Care. (2006) 29:1664–6. 10.2337/dc06-0631 16801598

[22] Ueland T Jemtland R Godang K Kjekshus J Hognestad A Omland T Prognostic value of osteoprotegerin in heart failure after acute myocardial infarction. J Am Coll Cardiol. (2004) 44:1970–6. 10.1016/j.jacc.2004.06.076 15542278

[23] Pedersen S Mogelvang R Bjerre M Frystyk J Flyvbjerg A Galatius S Osteoprotegerin predicts long-term outcome in patients with ST-segment elevation myocardial infarction treated with primary percutaneous coronary intervention. Cardiology. (2012) 123:31–8. 10.1159/000339880 22964478

[24] Bjerre M Munk K Sloth AD Nielsen SS Flyvbjerg A Bøtker HE. High osteoprotegerin levels predict MACCE in STEMI patients, but are not associated with myocardial salvage. Scand Cardiovasc J. (2014) 48:209–15. 10.3109/14017431.2014.917767 24758546

[25] Jono S Otsuki S Higashikuni Y Shioi A Mori K Hara K Serum osteoprotegerin levels and long-term prognosis in subjects with stable coronary artery disease. J Thromb Haemost. (2010) 8:1170–5. 10.1111/j.1538-7836.2010.03833.x 20230427

[26] Omland T Drazner MH Ueland T Abedin M Murphy SA Aukrust P Plasma osteoprotegerin levels in the general population: relation to indices of left ventricular structure and function. Hypertension. (2007) 49:1392–8. 10.1161/hypertensionaha.107.087742 17470718

