# Supplementary material for: Plasma osteoprotegerin predicts adverse cardiovascular events in stable coronary artery disease: the PEACE trial
Source: Front Cardiovasc Med. 2023 Jun 14;10:1178153. doi: 10.3389/fcvm.2023.1178153 (PMC10300416; doi:10.3389/fcvm.2023.1178153)
Supplement: Supplementary file 1 [file Datasheet1.docx]

**Supplementary Table 1** Pooled hazard risks of primary and secondary outcomes per 1-SD increase in lnOPG

|  | **HR (95% CI)** | **P value** |
| --- | --- | --- |
| **Primary Outcome** |  |  |
| Unadjusted HR (95% CI)  SD1  SD2  SD3  SD4  SD5 | Ref  0.97(0.34,2.82)  1.24(0.45,3.40)  1.59(0.58,4.36)  3.09(1.11,8.60) | \| 0.96 \| \| --- \| \| 0.68 \| \| 0.37 \| \| 0.03 \| |
| Adjusted HR (95% CI) ^#^ | 1.31(1.12,1.54) | 0.001 |
| **All-cause Death** |  |  |
| Unadjusted HR (95% CI)  SD1  SD2  SD3  SD4  SD5 | Ref  1.84(0.56,5.99)  2.72(0.86,8.57)  2.92(0.92,9.21)  5.31(1.66,16.98) | \| 0.32 \| \| --- \| \| 0.09 \| \| 0.07 \| \| 0.005 \| |
| Adjusted HR (95% CI) | 1.35(1.18,1.54) | <0.001 |
| **Cardiovascular Death** |  |  |
| Unadjusted HR (95% CI)  SD1  SD2  SD3  SD4  SD5 | Ref  0.86(0.25,2.96)  1.11(0.35,3.58)  1.22(0.38,3.95)  2.34(0.71,7.75) | \| 0.81 \| \| --- \| \| 0.86 \| \| 0.74 \| \| 0.17 \| |
| Adjusted HR (95% CI) | 1.28(1.04,1.56) | 0.02 |
| **HF Hospitalization** |  |  |
| Unadjusted HR (95% CI)  SD1  SD2  SD3  SD4  SD5 | Ref  1.68(0.22,13.13)  1.93(0.26,14.22)  3.07(0.42,22.44)  6.42(0.87,47.54) | \| 0.62 \| \| --- \| \| 0.52 \| \| 0.27 \| \| 0.07 \| |
| Adjusted HR (95% CI) | 1.38(1.08,1.76) | 0.01 |

# Adjusted for OPG, sex, estimated glomerular filtration rate (eGFR), total cholesterol, smoking, CCS functional classification, body mass index (BMI), history of hypertension and diabetes.

**Supplementary Table 2** Baseline OPG levels and risk of primary outcome and all-cause death stratified by different baseline medical history

|  | **HR (95%CI)** | **P Value** | **P for interaction** |
| --- | --- | --- | --- |
| **Primary Outcome** |  |  |  |
| Hypertension |  |  | 0.69 |
| Yes | 1.08(0.99,1.18) | 0.07 |  |
| No | 1.11(1.01,1.22) | 0.03 |  |
| Diabetes |  |  | 0.37 |
| Yes | 1.03(0.88,1.20) | 0.72 |  |
| No | 1.11(1.03,1.19) | 0.005 |  |
| Myocardial Infarction |  |  | 0.49 |
| Yes | 1.08(0.99,1.19) | 0.10 |  |
| No | 1.13(1.04,1.23) | 0.004 |  |
| Stroke |  |  | 0.84 |
| Yes | 1.12(0.95,1.31) | 0.19 |  |
| No | 1.10(1.03,1.17) | 0.007 |  |
| ACEI Treatment |  |  | 0.97 |
| Yes | 1.10(1.01,1.21) | 0.04 |  |
| No | 1.11(1.02,1.20) | 0.02 |  |
| **All-cause Death** |  |  |  |
| Hypertension |  |  | 0.93 |
| Yes | 1.09(1.01,1.17) | 0.03 |  |
| No | 1.09(1.00,1.18) | 0.05 |  |
| Diabetes |  | 0.90 | 0.24 |
| Yes | 0.99(0.85,1.16) | 0.002 |  |
| No | 1.1(1.04,1.17) |  |  |
| Myocardial Infarction |  |  | 0.79 |
| Yes | 1.08(1.00,1.17) | 0.04 |  |
| No | 1.1(1.02,1.19) | 0.02 |  |
| Stroke |  |  | 0.50 |
| Yes | 1.00(0.75,1.32) | 0.97 |  |
| No | 1.1(1.04,1.16) | 0.001 |  |
| ACEI Treatment |  |  | 0.60 |
| Yes | 1.07(0.99,1.17) | 0.10 |  |
| No | 1.11(1.03,1.19) | 0.006 |  |

**Supplementary Table 3** Baseline OPG levels stratified by ACEI treatment.

|  | OPG Median  (IQR 25%-75%) | P Value |
| --- | --- | --- |
| ACEI Treatment |  | 0.604 |
| Yes | 2.19(1.65,2.89) |  |
| No | 2.15(1.67,2.85) |  |
