# Supplementary figures and images for: Plasma osteoprotegerin predicts adverse cardiovascular events in stable coronary artery disease: the PEACE trial
Source: Front Cardiovasc Med. 2023 Jun 14;10:1178153. doi: 10.3389/fcvm.2023.1178153 (PMC10300416; doi:10.3389/fcvm.2023.1178153)

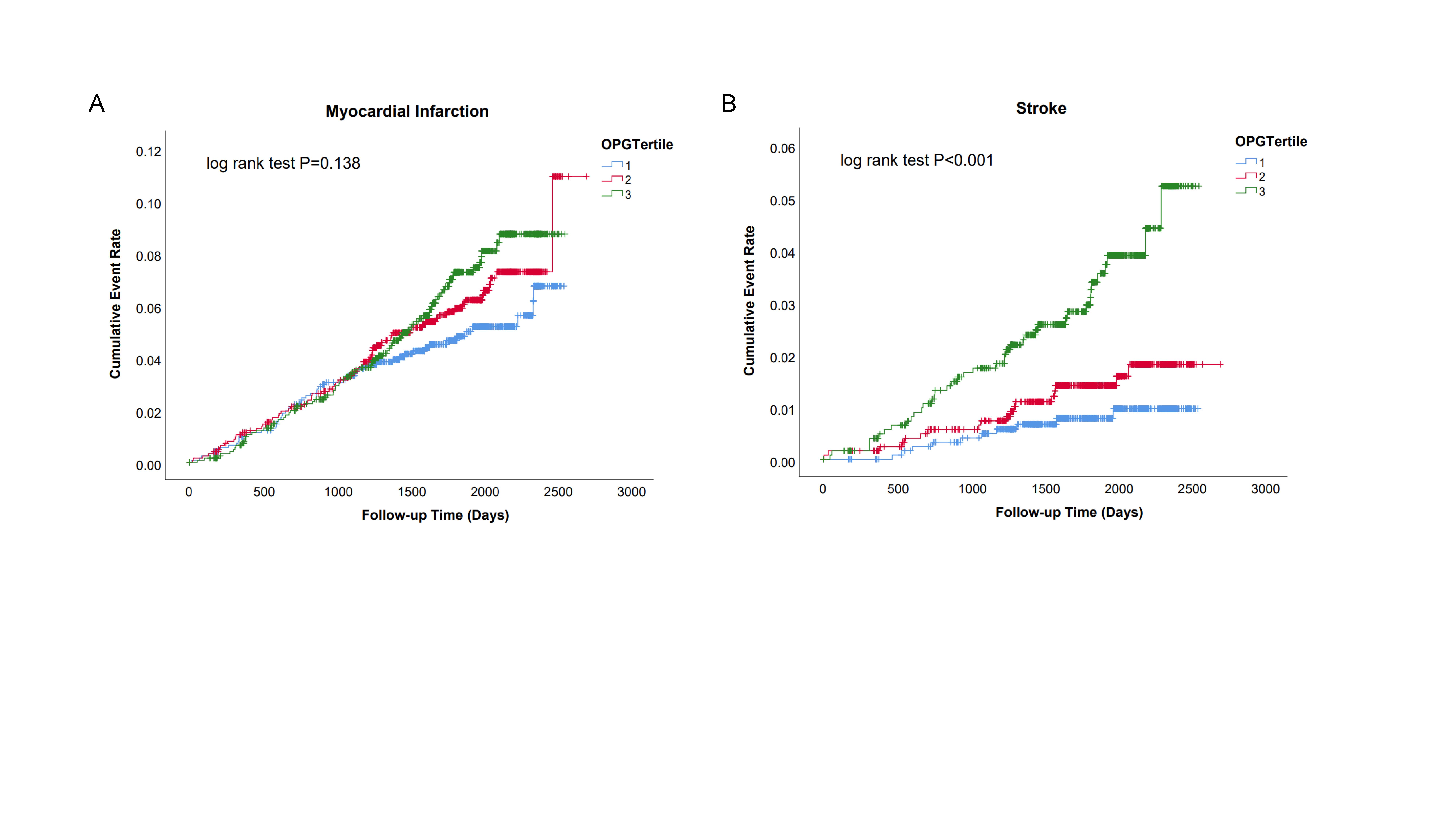

Supplement: Supplementary Figure 1 — Survival regarding the myocardial infarction (A) and stroke (B) stratified according to the tertile distribution of OPG. [file Image1.jpeg]

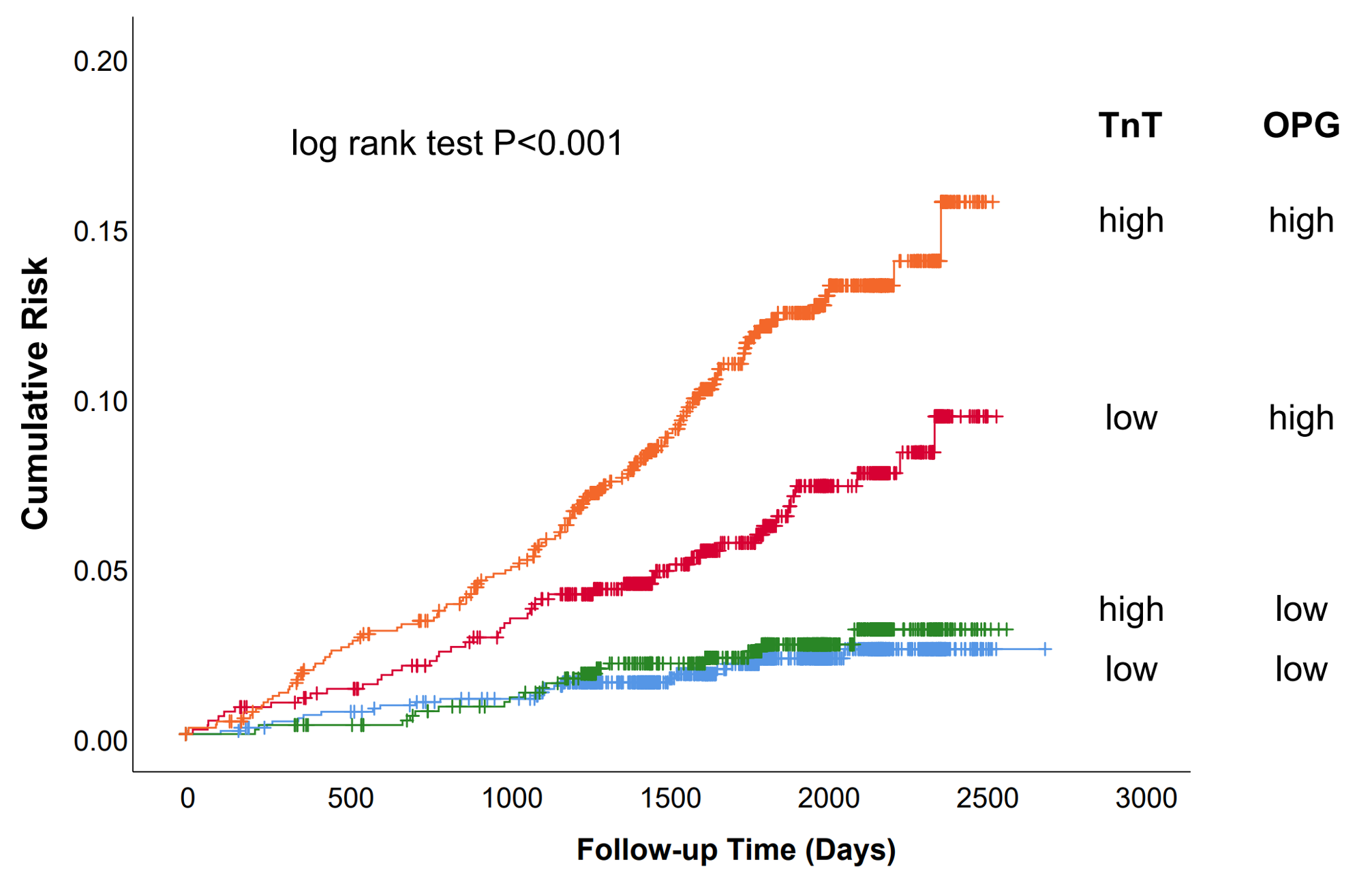

Supplement: Supplementary Figure 2 — Survival to the primary endpoint (cardiovascular death and congestive heart failure) stratified by median baseline levels of OPG and TnT. Kaplan-Meier analysis stratified by median baseline OPG and TnT levels. Baseline median OPG was 2.18 ng/mL; the median level for TnT was 5.97 ng/mL. “High” indicates values above the median and “low” values below the median. [file Image2.tiff]
